# BRCA1-associated protein 1 deficiency in lung adenocarcinoma predicts poor outcome and increased tumor invasion

**DOI:** 10.1186/s12885-016-2670-x

**Published:** 2016-08-23

**Authors:** Chen Shen, Yiqin Wang, Ping Wei, Xiang Du

**Affiliations:** 1 Department of Pathology, Fudan University Shanghai Cancer Center, Shanghai, 200032 China; 2 Department of Oncology, Shanghai Medical College, Fudan University, Shanghai, 200032 China; 3 Institute of Pathology, Fudan University Shanghai Cancer Center, No.270, Dong’an Road, Xuhui District, Shanghai, 200032 China; 4 Obstetrics and Gynecology Hospital of Fudan University, Shanghai, 200011 China

**Keywords:** BAP1, Lung adenocarcinoma, Invasion, Tumor suppressor

## Abstract

**Background:**

The major pathological type of non-small cell lung cancer is lung adenocarcinoma (LAC), which has a poor prognosis. BRCA1-associated protein-1 (BAP1) is a newly identified tumor suppressor that regulates a number of cellular functions in somatic malignancies. However, the impact of BAP1 expression in LAC has not been investigated.

**Methods:**

A total of 112 cases of LAC and 101 cases of non-neoplastic lung diseases were included in this study. The study focused on BAP1 expression in lung tissues and its relationship to patients’ clinical and pathological features. BAP1 expression was detected by immunohistochemistry. A human LAC cell line NCI-H1299 was transfected with lipofectamine p3xFLAG-BAP1. BAP1 gene expression was silenced in another LAC cell line NCI-H1650, in order to test the inhibitory effect of BAP1 on cell migration and invasion, as well as cell cycle regulation.

**Results:**

BAP1 expression showed a negative correlation with tumorigenesis of LAC (*p* <0.001) and lymph node metastasis (*p* = 0.010). High expression of BAP1 predicted longer disease free survival (*p* = 0.040) and overall survival (*p* = 0.021) of LAC patients. In functional assays, BAP1 was found to inhibit the migration and invasion of LAC cells, and promoted their apoptosis and necrosis.

**Conclusions:**

We identify BAP1 as a LAC precursor as well as a robust prognostic indicator in LAC patients. This study provides in vitro rationale for the further investigation of BAP1 in preclinical studies.

## Background

Lung cancer is the most common cause of cancer-related death with an annual mortality rate of 18 % worldwide [1]. Non-small cell lung cancer (NSCLC) presents with advanced pathologic features and has a poor prognosis. Lung adenocarcinoma (LAC) is currently the predominant histological subtype of NSCLC [2] with an average 5-year survival rate of 15 % despite using epidermal growth factor receptor (EGFR)-targeted therapies. This poor outcome is due in a large part to the delay in diagnosis in the early stages and a paucity of treatments against lymph node metastasis [3, 4]. Evidence also suggests that genetic susceptibility also contributes to malignancy risk in LAC [5].

Ubiquitin proteasome system (UPS) is involved in intracellular protein degradation, playing a crucial role in physiological processes including cell proliferation, apoptosis and migration [6, 7]. Deubiquitination enzymes (DUBs) act as hydrolases, catalyzing the reverse reaction of ubiquitination [8, 9]. So far, six subfamilies of DUBs have been found: the ubiquitin C-terminal hydrolases (UCHs), the ubiquitin-specific proteases (USPs/UBPs), the ovarian tumor proteases (OTUs), the Machado-Joseph disease protein domain proteases (MJDs), the Jab1/MPN domain-associated metalloisopeptidase (JAMM) domain proteins and the monocyte chemotactic protein-induced protein (MCPIP) [9, 10].

BRCA1-associated protein-1 (BAP1), is a 729aa protein and UCH family member, which exerts its DUB function via binding to the RING finger domain of BRCA1 thereby inhibiting its self-ubiquitination [11, 12]. Accumulating evidence has implicated BAP1 as a tumor suppressor since frequent loss of heterozygosity, large rearrangements, deletions and missense mutations of the *BAP1* gene have been found in breast tumors and lung cancer [13, 14]. Evidence has indicated that both DUB enzyme activity and nuclear localization are necessary conditions for its tumor suppressor activity [15, 16]. In functional experiments, BAP1 has been shown to suppress lung cancer tumorigenesis in athymic nude mice, promote the cell cycle and induce both apoptosis and necrosis. Moreover, BAP1-mediated regulation of cell growth is independent of wild-type BRCA1 [16]. In contrast, there is little research paper exploring the effect of BAP1 on tumor metastasis, particularly in lung cancer [17]. Recent research has suggested that BAP1 may be a prognostic factor in advanced NSCLC although this has not been thoroughly investigated [12].

In the present study we show that the deficiency in BAP1 expression is a strong predictor of malignant transformation of LAC cells. We also report a positive correlation between BAP1 and patient outcome and provide evidence that BAP1 overexpression promotes migration and invasion of adenocarcinoma.

## Methods

### Patients and tissue samples

A total of 112 cases of LAC and 101 cases of non-neoplastic lung diseases were collected in Fudan University Shanghai Cancer Center (Shanghai, China) from 2007 to 2011. Non-neoplastic lung diseases include a wide range of pathologic disorders from sclerosing hamartoma, pulmonary tuberculosis, pneumonia to bronchiectasis. Among the 112 cases of LAC, 59 were male and 53 were female, with an age range of 33 to 79 years (mean 57.6). Twelve out of 112 LAC cases were diagnosed as well-differentiated adenocarcinoma, 82 as showing moderate differentiation and the remaining 19 cases as poorly differentiated. According to the Union for International Cancer Control’s TNM staging criteria (2009), 58 patients were considered pathologic stage I or II and 54 with stage III, with no patient with stage IV undergoing surgery. The inclusion criteria were as follows: (a) LAC was confirmed by surgery and pathology; (b) use of bronchoscope specimens was excluded; (c) preoperative chemotherapy or radiotherapy was not received; (d) similar postoperative chemotherapy regimens were conducted. The median follow-up period for all patients was 25 months. Written informed consent was obtained from each patient.

### Immunohistochemistry

BAP1 immunohistochemical (IHC) staining was performed on all 213 surgical specimens from patients who were treated by pneumonectomy, lobectomy or wedge resection of lung. Formalin-fixed and paraffin-embedded tissues for IHC were dewaxed in xylene and dehydrated in ethanol. Sections were cut at 4-μm thickness, and then rinsed in 0.05 mol/L phosphate buffer solution (PBS) and incubated overnight at 4 °C with anti-BAP1 antibodies (1:200, HPA028814, Sigma, St. Louis, USA). We also employed 3 % hydrogen peroxide for the inhibition of endogenous peroxidase activity. Steps were performed using DAB horseradish peroxidase color development Kit (Sangon Biotech, Shanghai, China) according to the manufacturer’s instructions. We mixed slides of non-neoplastic disease and slides of LAC tissues so that in each batch of assay there were slides from both groups acting as positive and negative controls.

Brown nuclear staining of LAC cells or bronchial epithelial cells is considered positive. Evaluation of IHC staining was conducted on anonymized slides by two independent pathologists by light microscopy (Olympus Corp. BX-50). The labels of slides were carefully covered up so that none of the observers was able to make sure about the patient health information. Intensity of positive immunostaining was graded as low expression (<30) and high expression (≥30 %) [12] using 30 % as a cut-off point. To model on this method, I presuppose 10 as cut-off point and divide all patients into two groups, less than 10 positive and more than 10 % positive. Then, draw a survive curve of each group, calculate the *p*10 % value between the two groups. Second, I presuppose 20 % as cut-off point, divide all patients into two groups and calculate the *p*20 % value between them, and so forth. Then I get the value of *p*10 %, *p*20 %, *p*30 %, up to *p*90 %. Choose the minimum *p* value from the former nine numbers, so that the percentage is the ideal cut-off point. Therefore we instead use 30 % as cut-off point according to the literature. Images were captured from an Olympus Corp. Oly 760 video camera.

### Cell culture

The human LAC cell lines NCI-H1650, NCI-H1299, SPCA-1 and large cell lung cancer cell line NCI-H460 were purchased from American Type Culture Collection. Cells were initially maintained in RPMI-1640 medium (Gibco, U.S.A.), supplemented with 10 fetal bovine serum and 1 % penicillin and streptomycin, incubated at 37 °C in a humidified atmosphere containing 5 % CO_2_.

### Gene silencing, plasmid DNA construction and transfection

We measured the level of BAP1 background expression in the four lung cancer cell lines mentioned above by western blot. H460 was excluded because it originates in large cell undifferentiated carcinoma. The work on gene silencing was performed on H1650, which overexpresses the BAP1 protein, to obtain an artificial LAC cell line that expresses extremely low levels of BAP1. Meanwhile, we transfected the cell line H1299, which expressed the lowest level of background BAP1, with plasmid DNA to generate a cell line with artificially high expression of BAP1.

**Fig. 1:**
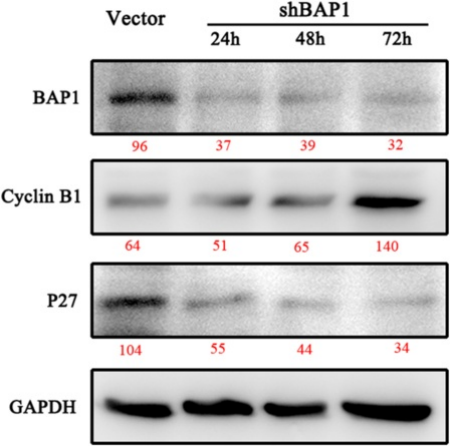
Confirmation of expression of BAP1 and cell cycle and growth-related proteins by Western blotting within 72 h

**Fig. 2:**
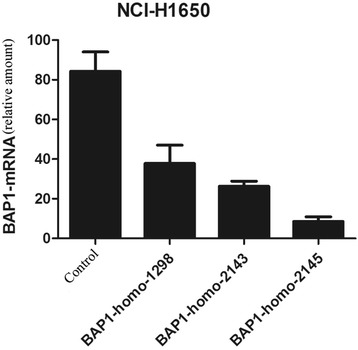
At the beginning, 3 si oligos of different sequence were ordered. The sequences are listed as Table 4. Then the silence effects of BAP1-siRNAs in H1650 cells were tested by Rt-PCR. Relative amount of BAP1-mRNA decreased to 49 (0.49 ± 0.21), 32 (0.32 ± 0.07) and 10 % (0.10 ± 0.03). The best silencing effect was interfered by BAP1-homo-2145. So we used si oligos of this sequence in the subsequent experiments

We used Lipofectamine 2000 (Invitrogen) for transfection of both siRNA and plasmid DNA, according to the manufacturer’s instructions. Transient knockdown was carried out with 100 nmol/L BAP1 siRNA or negative control (NC) siRNA (GenePharma, Shanghai, China) in H1650 as previously described. The effect of transient knockdown was proved lasting no less than 72 hours. (Fig. 1) We ordered three siRNA oligos of different sequence, all of whom showed an effective in silencing BAP1 expression in different extent, and selected the most efficient as assessed by RT-PCR (Fig. 2 and Table 1). H1650 cells (2.5 × 10^5^) were seeded into 6-well plates and incubated overnight, after which double-stranded siRNAs were transfected into cells. RNA or protein extraction was performed within 72 h. The sequences of NC siRNA pairs were: 5′-UUCUCCGAACGUGUCACGUTT-3′ (sense), and 5'-ACGUGACACGUUCGGAGAATT-3' (anti-sense). Sequences of BAP1 siRNA pairs were: 5'-GGCUGAGAUUGCAAACUAUTT-3' (sense), and 5'-AUAGUUUGCAAUCUCAGCCTT-3' (anti-sense). Full-length human BAP1 cDNA (GenBank accession number: NM_004656.3) was generated and cloned by polymerase chain reaction (PCR). The p3xFLAG-BAP1 vector was purchased from Bioworld Technology, Inc. (Shanghai, China). The amplified cDNA was sequenced to ensure there were no missense mutations. H1299 was then transfected with recombinant vectors.

**Table 1:** Sequences of 3 si oligos

siRNA serial	Sequence and antisense sequence
BAP1-homo-1298	
Sense (5′–3′)	CGG CCU UUC UAG ACA AUC ATT
Antisense (5′–3′)	UGA UUG UCU AGA AAG GCC GTT
BAP1-homo-2143	
Sense (5′–3′)	GAG GCU GAG AUU GCA AAC UTT
Antisense (5′–3′)	AGU UUG CAA UCU CAG CCU CTT
BAP1-homo-2145	
Sense (5′–3′)	GGC UGA GAU UGC AAA CUA UTT
Antisense (5′–3′)	AUA GUU UGC AAU CUC AGC CTT
Negative control (scramble RNA)	
Sense (5′–3′)	UUC UCC GAA CGU GUC ACG UTT
Antisense (5′–3′)	ACG UGA CAC GUU CGG AGA ATT
Positive control (GAPDH)	
Sense (5′–3′)	GUA UGA CAA CAG CCU CAA GTT
Antisense (5′–3′)	CUU GAG GCU GUU GUC AUA CTT

### RNA extraction and RT-qPCR

Total RNA was extracted from cultured LAC cells using TRIzol from Invitrogen. Quantitative analysis of BAP1 mRNA expression was performed by RT-qPCR and repeated three times for each batch. Primes for BAP1 RNA amplification were 5'-ATGAATAAGGGCTGGCTGGAGCTG-3' (forward) and 5'-GGGTATCAGCTGGTGGGCAAAGAA-3' (reverse). Thermal cycling conditions were as follows: one cycle of 95 °C for 5 min; 40 cycles for 95 °C for 20 s, 58 °C for 30 s, and 68 °C for 45 s; and one cycle of 72 °C for 10 min. The specificity of the PCR amplification was validated by a single peak in the melting curve.

### Western blot analysis

Total protein samples, lysed from cells using RIPA lysis buffer (Abcam), were separated using 10 % SDS-PAGE gel electrophoresis and then transferred onto a nitrocellulose membrane. The membrane was blocked with 5 % (w/v) Bovine serum albumin (BSA) in TBST (50 mM Tris–HCl pH 7.5, 150 mM NaCl, 0.1 % Tween-20), and incubated with primary antibody at 4 °C overnight followed by horseradish peroxidase-conjugated secondary antibody in 5 % BSA-TBST the next day for 2 h at room temperature. The immunoreactive bands were visualized using enhanced chemiluminescence with ECL luminescence (Thermo Scientific, Hudson, NH, USA).

Primary antibodies used were anti-GAPDH (#5632–1, Epitomics, Burlingame, CA, USA) as a loading control, anti-cyclinB1 (#4135, Cell Signaling, Boston, Massachusetts, USA), anti-P27 (kip1, #PA5–17830, Lab Vision Corporation, Fremont, CA) and anti-PARP-1 (#ab32138, Abcam, Massachusetts, USA).

### Cell proliferation assays

The proliferation of H1650 and H1299 cells transfected with siRNA or p3xFLAG-BAP1 was indirectly assayed using the Cell Counting Kit-8 (CCK-8, Dojindo, Japan), which quantifies cell viability. One hundred microliters of transfected cells (approximately 5 × 10^3^) were then plated into each well of a 96-well plate. At 24 h, 48 h, and 72 h, 10 μl of CCK-8 reagent was added to each well, and the cells were incubated at 37 °C for 2 h. The optical density (OD) at 450 nm was measured using an automatic microplate reader (Synergy4; BioTek, Winooski, VT, USA).

For the cell cycle assay, cells were seeded in 6-well plates (2.5 × 10^5^ cells per well) and harvested after 24 h of treatment with blank vector or plasmid DNA, followed by 12 h cultivation in serum-free medium. Cell cycle distribution was determined by flow cytometry using FACS Calibur (Becton-Dickinson and Company, Franklin Lakes, NJ, USA) with propidium iodide (PI) and analyzed using CELL Quest software (Becton Dickinson and Company, CA, US.).

Apoptosis was assessed using the Annexin V-FITC apoptosis detection kit I and FITC-conjugated antibody was purchased from the Beyotime Institute of Biotechnology (Shanghai, China). Cells were seeded in 6-well plates, incubated with doxorubicin (1 μl per well) for 16 h, and harvested as previously described. DNA content was measured after 30 min of staining with PI. Finally, the cells were analyzed with a flow cytometer using an Epics system (Coulter Epics XL) equipped with an argon ion laser operated at a wavelength of 488 nm. Surface exposure of phosphatidylserine in apoptotic cells was measured using the Annexin V-FITC/PI apoptosis detection kit I.

### Wound healing assay and Transwell assay

For the wound healing assay, H1650 cells and H1299 cells were grown until 70 % confluent in 6-well plates. Then H1650 cells were transfected with siNC or siBAP1, while H1299 cells were treated with blank vector or p3xFLAG-BAP1 overnight. Cells were scraped off using a pipette tip and washed with PBS twice to remove floating cells and debris. Representative images of cells migrating into the wounds were captured at 0 h, 24 h and 48 h in the same wounded region.

For cell invasion assays, Transwell inserts with an 8-μm pore size for 24-well plates were pre-coated with Matrigel (ECM550, Chemicon). Transfected cells were seeded in the upper chamber (8 × 10^4^ cells per well) in RPMI 1640 serum-free medium. Medium with 20 % FBS was added in the bottom chamber. After 24 h (for H1299 cells) or 48 h (for H1650 cells), the cells on the upper surface of the filter were removed by wiping with a cotton swab, and the invasive cells that penetrated through the Matrigel were counted in 20 random grids under an inverted microscope (40×).

### Statistical analysis

The statistical significance of the correlation between expression of BAP1 and several clinicopathological parameters was assessed by the chi-squared (*χ*^2^) test, as was the correlation between BAP1 expression and malignant status. The probability of cumulative overall survival (OS) and disease free survival (DFS) as a function of time was estimated by the Kaplan–Meier method and the log-rank test. Multivariate survival analysis was performed using the Cox regression model. *P* values less than 0.05 were considered as statistically significant. SPSS (version 18.0, SPSS, Inc., Chicago, IL) was used for statistical evaluation. Histograms were constructed by Graphpad prism 5. The MW value was detected by ImageJ (Ver 1.48). The comparison of the two groups in the Transwell assay and RT-qPCR was verified by a *t* test.

## Results

### BAP1 expression in lung adenocarcinoma and clinicopathologic findings

**Fig. 3:**
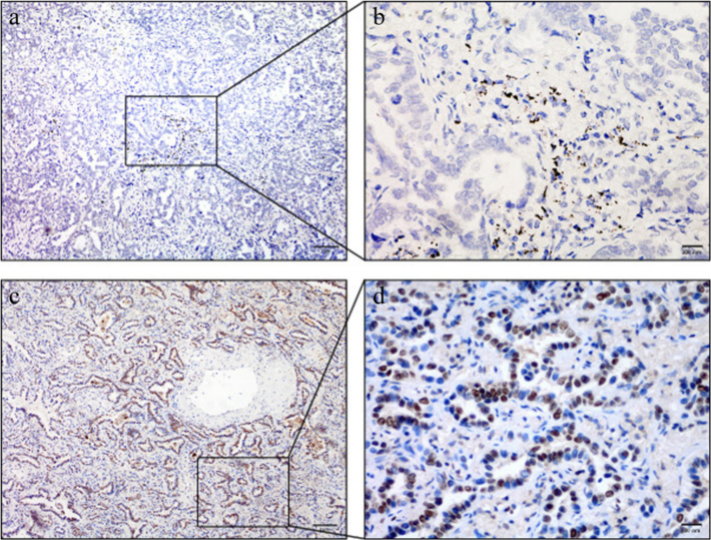
Immunohistochemical staining patterns of BRCA1-associated protein (BAP1) in lung adenocarcinoma. The upper two pictures (**a**, **b**) show low expression of BAP1 while the lower two (**c**, **d**) show high expression of BAP1. The cut-off point was 30 %. Scale bars are showed at the bottom right corner of each graph. Magnification is × 100 (**a**, **c**), and × 400 (**b**, **d**)

To investigate the potential role of BAP1 in the generation and progression of LAC, we performed immunohistochemical staining on samples from 112 cases of LAC and 101 cases of non-neoplastic lung diseases. BAP1 straining was strongly positive in 91 of the 101 non-neoplastic cases (90.1 %), significantly higher than the rate of high expression in LAC (32 out of 112 cases, 28.6 %; *p* < 0.01), suggesting a deficiency of BAP1 expression in LAC tissues (Fig. 3 and Table 2).

**Table 2:** Correlation between BAP1 expression and oncogenesis

Tissue	BAP1 expression		*P* value
	Low^a^	High^b^	
Non-neoplastic	10	91	<0.001
Malignant	80	32	

^a^ < 30 % bronchial epithelial cells or tumor cells were positive stained

^b^ ≥ 30 % bronchial epithelial cells or tumor cells were positive stained

Next we conducted a statistical analysis to explore the correlation between BAP1 expression and the clinicopathologic characteristic of LAC patients. Expression of BAP1 was closely related to tumor status (*p* = 0.044), pTNM stage (*p* = 0.007), node status (*p* = 0.010), pleural invasion (*p* = 0.004) and post-operation relapse or metastasis (*p* = 0.0028) (Table 3). However, there was no significant association between BAP1 expression and other clinicopathologic features, including patient sex, age, gross type, diameter of tumor in the greatest dimension, and degree of histological differentiation.

**Table 3:** Correlation between BAP1 expression and clinicopathologic parameters of lung adenocarcinoma

Clinicopathological features		n	%	BAP1 Expression		*P* value
				Low	High	
All cases		112	100	80	32	
Sex	Male	59	52.68	45	14	0.231
	Female	53	47.32	35	18	
Age (years) (Average 57.6)	<60	66	58.93	50	16	0.224
	≥60	46	41.07	30	16	
Gross Type	Central	82	73.21	59	23	0.840
	Peripheral	30	26.79	21	9	
Diameter of Tumor (cm) (Average 3.67)	<3.0	37	33.04	26	11	0.849
	≥3.0	75	66.96	54	21	
Differentiation	Well	12	10.71	7	5	0.458
	Moderate	82	73.21	61	21	
	Poor	18	16.07	12	6	
Tumor Status	T1, T2	97	86.61	66	31	**0.044**
	T3, T4	15	13.39	14	1	
pTNM Stage	I, II	58	51.79	35	23	**0.007**
	III	54	48.21	45	9	
Node Status	N0, N1	59	52.68	36	23	**0.010**
	N2, N3	53	47.32	44	9	
Pleural Invasion	(−)	43	38.39	24	19	**0.004**
	(+)	69	61.61	56	13	
Post-operative relapse or metastasis	(−)	45	40.18	27	18	**0.0028**
	(+)	67	59.82	53	14	

*P* value, below 0.05 threshold, is in bold

### Survival analysis

**Fig. 4:**
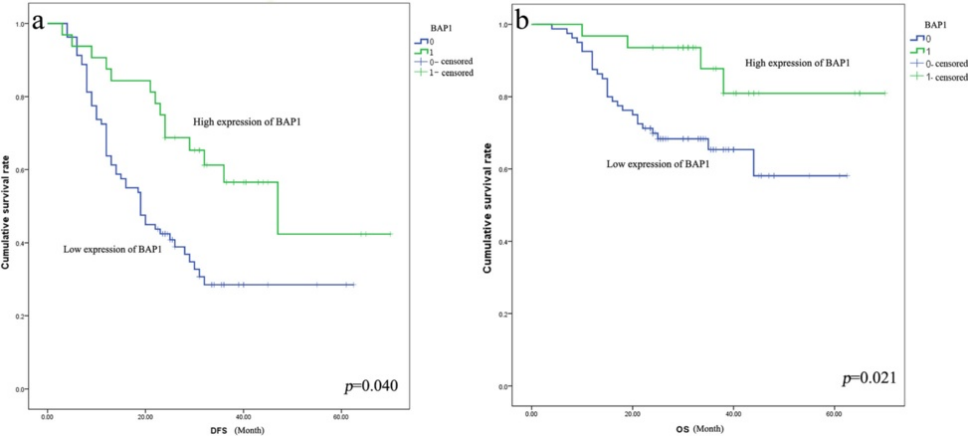
Correlation between BAP1 expression and disease free survival/Overall Survival FS/OS. Curve (**a**) shows that BAP1 expression was significantly associated with disease free survival (*p* = 0.040). And curve (**b**) shows that BAP1 predicts a longer overall survival in lung adenocarcinoma (LAC) (*p* = 0.021)

To determine the association between BAP1 expression and patient outcome as measured by OS and DFS, we performed Kaplan–Meier analysis and log-rank test after 25 months of follow-up. As shown in Fig. 4, higher expression of BAP1 in LAC predicted a higher OS rate and DFS rate (*p* = 0.040 and *p* = 0.021, respectively). A subsequent multivariate survival analysis of prognostic factors again demonstrated that higher expression of BAP1 was an independent prognostic factor in LAC, significantly associated with a longer life expectancy (Fig. 4 and Table 4). Notably, pTNM stage, another independent prognostic factor of LAC, also showed a significant association with BAP1 expression (Tables 3 and 4), suggesting that high expression of BAP1 strongly predicts longer survival and better prognosis in LAC.

**Table 4:** Multivariate Cox regression model for lung adenocarcinoma patients’ survival

Characteristics	*P* value	SE	Exp(B)	95 % CI for Exp(B)
BAP1 expression	0.185	0.578	0.465	0.150–1.444
Pleural Invasion	**0.048**	0.479	2.584	1.010–6.608
Differentiation	**0.003**	0.365	2.930	1.433–5.990
TNM Stage	**0.027**	0.383	2.329	1.099–4.935

*P* value, below 0.05 threshold, is in bold

### BAP1 inhibits migration and invasion abilities of cells in vitro

**Fig. 5:**
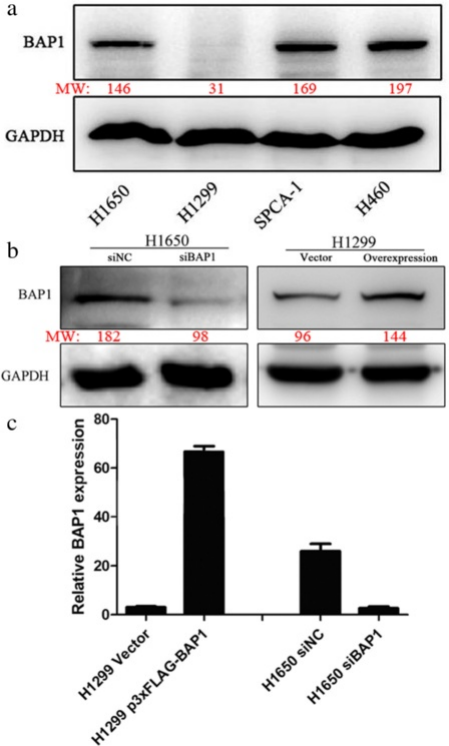
**a** Background expression of BAP1 in lung cancer cell lines. LAC cell lines H1650 and H460 expressed a high background BAP1, therefore one of these (H1650) was chosen for gene knockdown experiments. H1299 expressed the lowest BAP1, so was chosen for BAP1 overexpression experiments by plasmid transfection. Transfection efficiencies were detected by immunoblotting. **b** and Rt-PCR (**c**). MW is marked in red under the bands

**Fig. 6:**
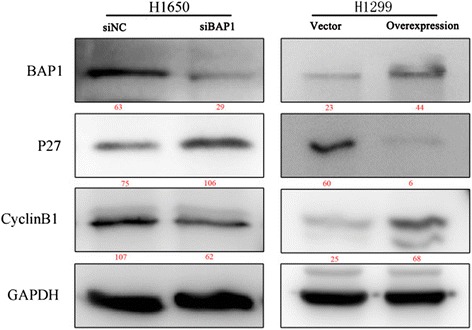
Expression of BAP1 and cell cycle-related proteins. P27 was inversely influenced by BAP1 while CyclinB1 expression changed in accordance with BAP1. It is widely accepted that the expression of P27 is down-regulated when the cell cycle runs slower. MW is marked in red under the bands

Based on the findings above, we hypothesized that BAP1 might function as an invasion suppressor protein in cells. We therefore constructed a p3xFLAG-BAP1 plasmid and siRNAs and evaluated their efficiency on lung cancer cell lines. An immunoblotting assay (IB) was adapted to detect baseline expression of BAP1 in four lung cancer cell lines, H1299, H1650, H1299 and H1650, to determine the best lines for subsequent functional experiments. H1299 cells showed the lowest level of BAP1 and H1650 cells showed the highest expression (Fig. 5). These were therefore selected for BAP1 enhancement and knockdown, respectively. H1299 cells were transfected with plasmid p3xFLAG-BAP1 for 48 h and H1650 cells were transfected with siBAP1 for 24 h followed by Real-time PCR and IB assay. The expression levels of both mRNA and protein were elevated after transfection in H1299 cells, yet suppressed in H1650 cells, indicating high efficiency of obtaining both siRNAs and recombinant plasmid (Fig. 6), as expected.

**Fig. 7:**
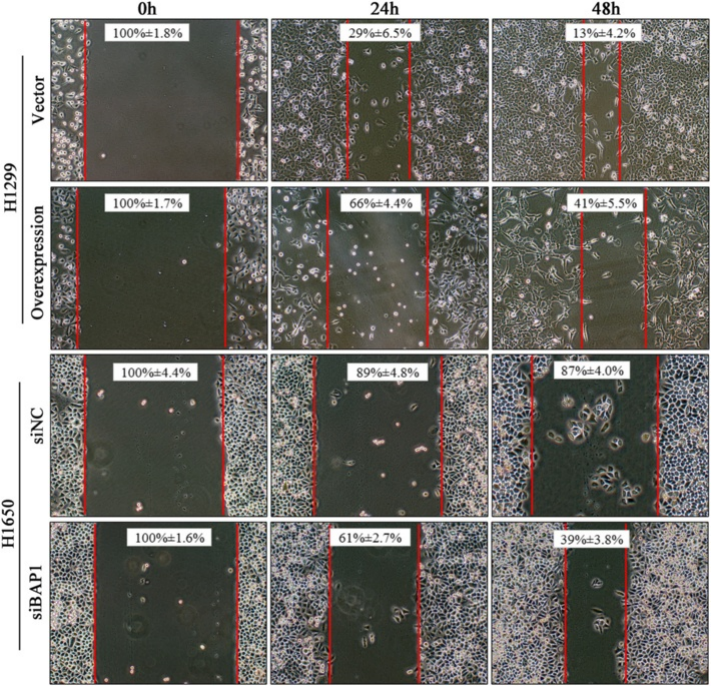
Wound-healing assay. From a baseline taken at 0 h, we measured the gap between the cells at 24 h and 48 h, which were recorded as percentages ± standard deviation. Width of the gap was compared with the control group, and compared by paired *t* test. *p < 0.05

**Fig. 8:**
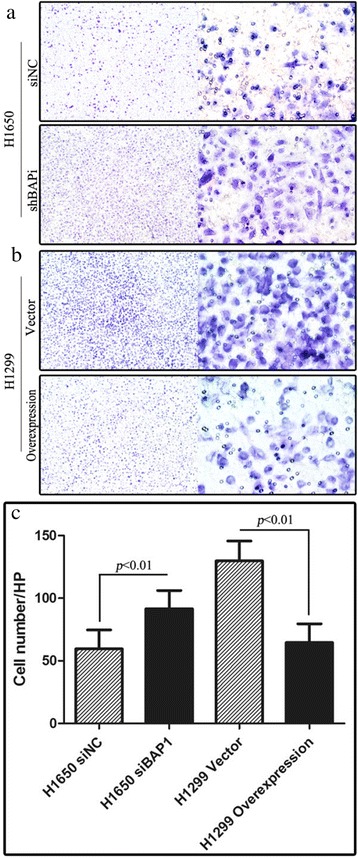
Transwell assay. The upper two graphs (**a**, **b**) shows cells that passed through Matrigel into the lower chamber. Magnification is × 100 (left), and × 400 (right). The average number of H1650 siNC cells that passed through Matrigel was 59.53 per field of view at high magnification, compared with 91.56 in H1650 siBAP1 (*p* < 0.01) **a**. The average number of H1299 vector cells that passed through Matrigel was 129.8 per FOV at high magnification, compared with 64.62 in H1299 cells overexpressing BAP1 (*p* < 0.01) **b**. The lower bar chart shows the number of invaded cells assessed by *t* test (**c**)

**Fig. 9:**
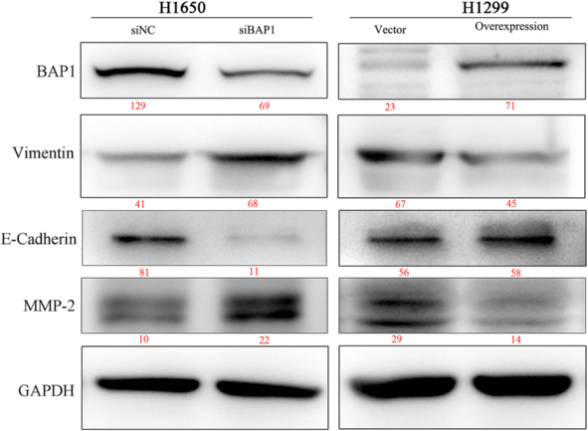
Synchronous detection of BAP1 expression and EMT proteins. The amount of vimentin varied in accordance with BAP1 while both MMP2 and E-Cadherin were inversely modulated with BAP1 expression

We performed cell wound-healing and Transwell in vitro cell invasion assay to investigate the functional role of BAP1. H1299 cells were transfected with p3xFLAG-BAP1 plasmid and H1650 cells were transfected with siBAP1. After 48 h, the cells were either subjected to the wound-healing assay or to the Transwell invasion assay. Cellular migration ability of H1299 was potently attenuated by BAP1 overexpression, meanwhile, BAP1 gene expression silencing in H1650 cells resulted in a remarkably faster wound healing process compared with the controls (Fig. 7). The Transwell invasion assay also showed that overexpression of BAP1 was accompanied with significantly attenuated cellular invasive ability in H1299, which, conversely, was enhanced in H1650 cells by silencing of siBAP1 (Fig. 8). Finally, to elucidate the mechanism by which BAP1 suppresses invasion and metastasis in LAC cells, we analyzed the expression levels of metastasis-related proteins in transfected H1299 cells and H1650 cells using IB, respectively. We observed a significant reduction of MMP-2 and Vimentin, as well as apparent increasing of E-Cadherin. One may therefore speculate that BAP1 affects the expression of metastasis-related proteins in these lines, which, in vivo, has the potential to influence the metastatic capability of LAC cells (Fig. 9).

### BAP1 promotes cell death and suppresses cell proliferation in LAC

**Fig. 10:**
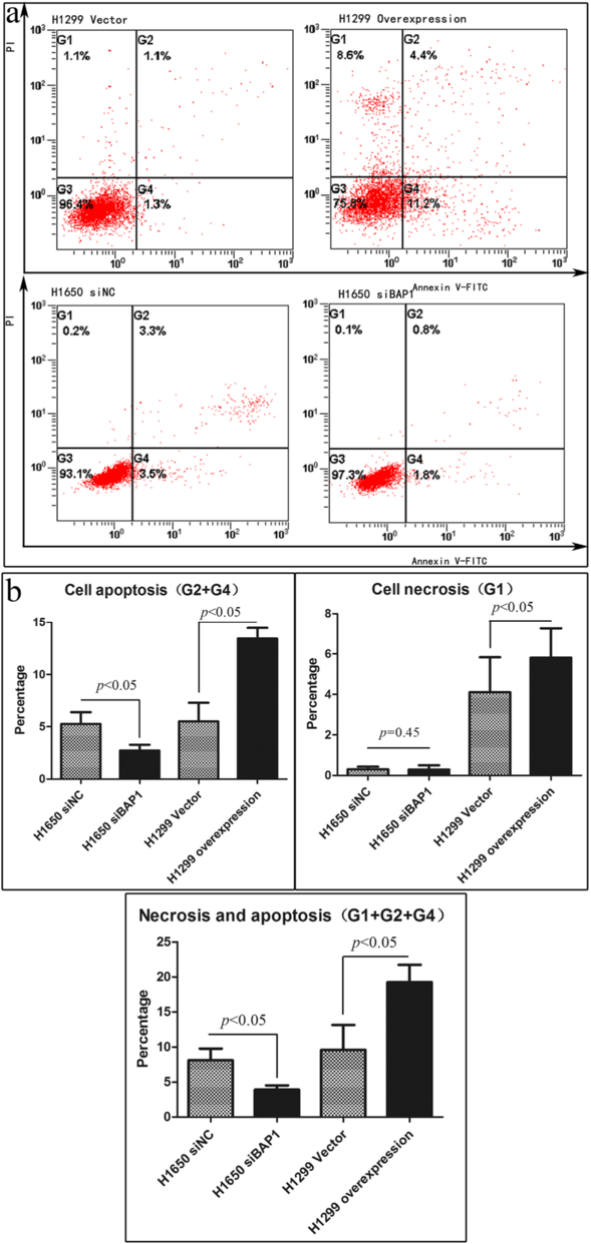
BAP1-mediated cell death displays properties of both apoptosis and necrosis. **a** Lung adenocarcinoma cells in early apoptosis (G4) were detected by flow cytometry. **b** Bar chart showing the percentage of early apoptosis cells

**Fig. 11:**
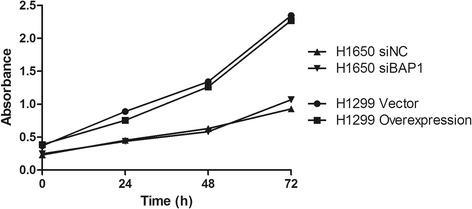
Correlation between BAP1 expression and cell proliferation expressed as cell proliferation curves

To address whether BAP1 also regulates cell death both by apoptosis and necrosis, we conducted a cell apoptosis assay in LAC cells. H1650 and H1299 cells, transfected with or without siBAP1 or BAP1 plasmid were incubated with doxorubicin for 16 h, before analysis by flow cytometry (FACS). Dual parametric dot plots combining Annexin-V and PI fluorescence show four quadrants, among which the lower left quadrant represented total viable cell population (Annexin-V^−^/PI^−^), the lower right represented the early stage apoptotic cell population (Annexin-V^+^/PI^−^), and the late necrotic and late apoptotic cells would cluster in the upper right-hand quadrant (Annexin-V^+^/PI^+^). We observed that overexpression of BAP1 gave rise to a sharp increase in early stage apoptotic population up to 13.45 % and a slight increase in late-stage apoptotic/necrotic cells up to 5.81 %, which was significantly higher than the control group (5.51 % apoptosis, *p* < 0.05; 4.11 % necrosis, *p* < 0.05). Conversely, silencing BAP1 expression reduced the early stage apoptotic cell population to 2.71 %, significantly lower than that of the control (5.25 %, *p* < 0.05), suggesting that BAP1 promotes tumor cell death by apoptosis and necrosis (Fig. 10). A set of cell proliferation assays was therefore performed to elucidate whether BAP1 also plays an essential role regulating cell proliferation. H1299 cells were transfected with BAP1 plasmid and H1650 with siBAP1 followed by treatment with CCK-8. The intersection point of the proliferation curves of siBAP1 H1650 cells and the control group was seen at 48 h, while the growth of BAP1-overexpressing H1299 cells almost overlapped that of the controls. The latter observations suggest that BAP1 has little effect on cell proliferation in LAC (Fig. 11).

## Discussion and conclusions

Study of BAP1 expression in malignant and benign lung tissues provides novel insights into the role of this protein in LAC. As a newly identified biomarker, BAP1 is highly expressed in non-neoplastic lung tissues and reversely correlated with tumor invasion and node metastasis. Moreover, BAP1 predicts longer survival in patients with LAC. Functionally, BAP1 promotes apoptosis and necrosis, and down-regulates the migration and invasion abilities of LAC cells. Thus we confirm that BAP1 serves as a cancer precursor as well as a powerful predictor of improved outcome and reduced likelihood of recurrence.

In the present study, we observed that BAP1 expression was significantly less frequent in LAC tissue (28.6 %) than in non-neoplastic lung diseases (90.1 %; Fig. 3; Table 2). This suggests that BAP1 might suppress initial lung tumorigenesis through an unknown mechanism, implying that further experiments on the role of BAP1 in cell apoptosis are required. We subsequently observed that BAP1-mediated cell death displays features implicating both apoptosis and necrosis. Thus, BAP1 might suppress tumorigenesis by facilitating tumor cell death by activation of both these pathways.

Our immunohistochemical and clinical investigations demonstrated that BAP1 expression was inversely correlated with pathologic TNM stage, node status, pleural invasion, rate of relapse and metastasis. According to the TNM classification for lung cancer of 2009, T stage depends on a range of factors including the maximum diameter of the tumor, pleural invasion, distant to the carina, atelectasis or obstructive pneumonitis, nerve invasion and separate tumor nodule(s) [18]. However, a small proportion of cases present with the following characteristics: (a) tumor in the main airway, less than 2 cm from the carina (none in the present study); (b) tumor accompanied by pneumonitis (3 cases); (c) separate tumor nodule(s) (5 cases). In these cases, tumor size as well as pleural invasion determine T stage to a large degree. Considering that BAP1 expression is not associated with tumor size (*p* = 0.849) but inversely correlated with pleural invasion (*p* = 0.004) (Table 3), it is reasonable to believe that pleural invasion had the greatest contribution. It is widely accepted that metastasis is a major hallmark of malignancy and cancer cell invasion is one of the essential early events in metastasis. BAP1 may inhibit the progression of LAC by attenuating tumor invasiveness, a function that was supported by the findings of the Transwell assay (Fig. 8). However, BAP1 is closely related to lymph node status (Table 3), suggesting that BAP1 also mediates suppression of cell migration in LAC which is in keeping with the findings of the wound-healing assay (Fig. 7).

The secretion of proteases such as matrix metalloproteinases (MMPs), capable of degrading the extracellular matrix (ECM) to create space through which cells can migrate, is also modulated by BAP1 expression. Specifically, overexpression of certain MMPs, including MMP2, is directly involved in the progression, invasion and metastasis of LAC [19–21]. E-Cadherin plays an essential role in the maintenance of the normal structure and adhesion of cells and its deregulation is associated with tumor invasion and metastasis, being reduced or absent in a vast majority of NSCLC cases [22–24]. Other tumor metastasis-associated proteins, Vimentin for example, are also down-regulated following the silencing of BAP1 [25]. According to the above results, we postulate that BAP1 may regulate these proteins and impact on tumorigenesis, inhibiting invasion and local metastasis.

Previous research has shown BAP1 to be an independent prognostic factor in colorectal carcinoma [26] and renal-cell carcinoma [27, 28]. This study reveals for the first time that BAP1 is associated with post-operation survival time of LAC, predicting longer OS and DFS. Further prospective studies of large cohorts are necessary to confirm the potential value of BAP1 in predicting the outcome of LAC patients before being put it into clinical practice.

## Abbreviations

BAP1, BRCA1-associated protein-1; BRCA1, breast cancer 1; BSA, bovine serum albumin; CCK-8, Cell Counting Kit-8; DFS, disease free survival; DUB, Deubiquitination enzyme; ECM, extracellular matrix; EGFR, epidermal growth factor receptor; FACS, fluorescence activated cell sorting; IHC, immunohistochemical; JAMM, Jab1/MPN domain-associated metalloisopeptidase; LAC, lung adenocarcinoma; MCPIP, monocyte chemotactic protein-induced protein; MJD, Machado-Joseph disease; MMP, matrix metalloproteinases; NC, negative control; NCI, National Cancer Institute; NSCLC, non-small cell lung cancer; OS, overall survival; OUT, ovarian tumor proteases; PBS, phosphate buffer solution; PI, propidium iodide; RT-PCR, real-time polymerase chain reaction; UCH, C-terminal hydrolase; UPS, Ubiquitin proteasome system; USP, Ubiquitin-specific proteases
